# Therapeutic Modulation of Arginase with nor-NOHA Alters Immune Responses in Experimental Mouse Models of Pulmonary Tuberculosis including in the Setting of Human Immunodeficiency Virus (HIV) Co-Infection

**DOI:** 10.3390/tropicalmed9060129

**Published:** 2024-06-06

**Authors:** Sadhana Chauhan, Rebecca J. Nusbaum, Matthew B. Huante, Alex J. Holloway, Mark A. Endsley, Benjamin B. Gelman, Joshua G. Lisinicchia, Janice J. Endsley

**Affiliations:** 1 Department of Microbiology and Immunology, University of Texas Medical Branch, Galveston, TX 77555, USA; sachauha@utmb.edu (S.C.); rjnusbaum@uchicago.edu (R.J.N.); m.huante@medpace.com (M.B.H.); alexholloway@mesogen.com (A.J.H.); maendsle@utmb.edu (M.A.E.); 2 Department of Pathology, University of Texas Medical Branch, Galveston, TX 77555, USA; bgelman@utmb.edu (B.B.G.); jglisini@utmb.edu (J.G.L.)

**Keywords:** arginase inhibitor, N^ω^-hydroxy-nor-L-arginine (nor-NOHA), polyamine, TB, HIV co-infection, immune responses

## Abstract

L-arginine metabolism is strongly linked with immunity to mycobacteria, primarily through the antimicrobial activity of nitric oxide (NO). The potential to modulate tuberculosis (TB) outcomes through interventions that target L-arginine pathways are limited by an incomplete understanding of mechanisms and inadequate in vivo modeling. These gaps in knowledge are compounded for HIV and Mtb co-infections, where activation of arginase-1 due to HIV infection may promote survival and replication of both Mtb and HIV. We utilized in vitro and in vivo systems to determine how arginase inhibition using N^ω^-hydroxy-nor-L-arginine (nor-NOHA) alters L-arginine pathway metabolism relative to immune responses and disease outcomes following Mtb infection. Treatment with nor-NOHA polarized murine macrophages (RAW 264.7) towards M1 phenotype, increased NO, and reduced Mtb in RAW macrophages. In Balb/c mice, nor-NOHA reduced pulmonary arginase and increased the antimicrobial metabolite spermine in association with a trend towards reduced Mtb CFU in lung. In humanized immune system (HIS) mice, HIV infection increased plasma arginase and heightened the pulmonary arginase response to Mtb. Treatment with nor-NOHA increased cytokine responses to Mtb and Mtb/HIV in lung tissue but did not significantly alter bacterial burden or viral load. Our results suggest that L-arginine pathway modulators may have potential as host-directed therapies to augment antibiotics in TB chemotherapy.

## 1. Introduction

*Mycobacterium tuberculosis* (Mtb), the causative of tuberculosis (TB), is the leading cause of deaths worldwide from a single infectious agent [1]. People living with HIV (PWH) or suffering from conditions that impair the immune system (under-nutrition, metabolic disturbance, alcohol use disorder, smoking, etc.) are more susceptible to Mtb infection [2,3,4,5,6,7]. The risk of developing active TB is significantly higher in PWH and the likelihood of worsened TB outcomes is also greater [8,9]. The emergence of multi-drug-resistant and XDR- TB, which are linked with the HIV pandemic, has also contributed significantly to increased efforts to control Mtb infections [10]. The relationship between poor immune status and TB control underscores the need to expand our understanding of immunity and develop host-directed therapies (HDTs) that will support antibiotic treatments.

As the primary host target for Mtb infection and a critical regulator of both innate and acquired immune responses, modulation of macrophage (Mϕ) responses through intervention offers significant therapeutic potential [11]. Activated Mϕ can control and eliminate Mtb, while Mtb manipulates the macrophage for survival [12] through complex and incompletely understood host-and-pathogen interactions. L-arginine metabolism plays an important role in the immune response of Mϕ to Mtb and other pathogens. The production of NO, resulting from the catabolism of L-arginine by inducible nitric oxide synthase (iNOS), has an important role in host immunity to Mtb, including in antibacterial activity, through nitrosative stress and regulation of inflammation [13,14,15,16,17]. Elevated levels of iNOS have been reported in human tuberculosis [18] and *nos2* knockout (KO) mice have rapidly progressing disease compared to WT counterparts following Mtb infection [19,20]. IFN-γ knockout mice that are infected with Mtb fail to produce reactive NO intermediates and are unable to restrict growth of bacteria [21,22,23]. The in vivo exacerbation of active or reactivation of latent TB with iNOS inhibitors [24,25] further demonstrate the role of NO and highlight the potential to modulate innate immunity against Mtb through therapeutic modalities that regulate NO in myeloid cells.

Arginase is a competitive inhibitor of iNOS, competing for their shared substrate L-arginine. Arginase metabolism of L-arginine promotes production of L-ornithine, proline and polyamines. The balance of iNOS and arginase in host macrophages is associated with well-defined Mϕ polarization states where inflammatory M1 and regulatory M2 Mϕ demonstrate an iNOS and Arg-1 bias, respectively [26]. It has demonstrated roles in immune modulation, cardiovascular function, and response to infectious diseases. Increased levels of arginase, as well as the upstream agonist IL-10, are found in peripheral blood mononuclear cells (PBMC) in HIV-seropositive patients and correlate with disease severity [27,28]. Roles for polyamines produced downstream of arginase activity have been described in both HIV and Mtb proliferation [27,29].

The properties of arginase as a potent competitive inhibitor of iNOS suggest the potential to modulate protective Mϕ function in the setting of TB through therapeutic intervention. The development of specific arginase inhibitors has been extensively reviewed by Pudlo M et al. [30]. Arginase inhibitors such as 2(S)-amino 6-boronohexanoic acid (ABH) and S-(2-boronoethyl)-L-cysteine (BEC) have shown to be potential therapies for treating several NO-dependent smooth-muscle disorders using pure arginase enzyme [31]. An improvement was reported in excisional wound healing when mice were topically treated with the arginase inhibitor ABH, suggesting possible applications in early wound closure. Two novel synthetic arginase inhibitors, SHK242 and SHK277, were identified to protect against allergen-induced early asthmatic reactions (EARs) and late asthmatic reactions (LARs), allergen-induced hypersensitive responsiveness (AHR), and lung inflammation in vitro [32]; however, in vivo, the novel compounds did not inhibit AHR or lung inflammation in a guinea pig model.

Among several synthetic arginase inhibitors studied, nor-NOHA has shown promising results in animals and in vitro models of parasitic and bacterial infection. Inhibition of arginase in vivo by nor-NOHA after infection with two species of *Leishmania* delayed, but did not prevent, the development of leishmaniasis. Arginase activity was reduced significantly with a corroborating increase of NO, suggesting the potential to control *Leishmania* infection in Balb/cJ mice by reducing parasite proliferation and heightening parasite killing from NO generation [33]. Modulation of arginase activity during infection with *Salmonella* reduced the bacterial burden and delayed death in a BALB/c disease model [34,35].

To date, translational studies to evaluate the therapeutic potential of arginase inhibition in animal models of experimental TB or TB/HIV co-infection are lacking. Therefore, we evaluated in vitro and in vivo outcomes of arginase inhibition using nor-NOHA in a murine Mϕ cell line and both BALB/cJ and human immune system (HIS) mouse models that we developed to understand the pathogenesis of Mtb and coinfection of HIV/Mtb [36,37,38,39]. These outcomes suggest that arginase inhibition approaches may hold promise to improve TB disease outcomes as host-directed therapies accompanying antibiotic chemotherapy but require further optimization through improved bioavailability or pathway complementation.

## 2. Materials and Methods

### 2.1. Mice, Cell Lines, Human Tissues

Balb/cJ mice and NOD.Cg-Prkdc^scid^Il2rg^tm1Wjl/^SzJ mice were purchased from Jackson Laboratory. Human immune system mouse (HIS mouse) was generated as described previously [38]. RAW 264.7 murine macrophage cell line was obtained from American Type Culture Collection (ATCC) and cultivated using DMEM media supplemented with 10% FBS, 1% penicillin/streptomycin, 1% L-glutamine, and 1% non-essential amino acids. Frozen specimens of HIV+ and age matched HIV- human lung were obtained from the National NeuroHIV Tissue Consortium (NNTC) [40].

### 2.2. Arginase Inhibitor

N^ω^-hydroxy-nor-L-arginine (nor-NOHA) (Cat#F-3685.0050) was purchased from Bachem AG, Bubendorf, Switzerland, as lyophilized powder. A stock solution (50 mg/mL) prepared in DMSO was stored at −20 °C in aliquots.

### 2.3. Bacteria and Virus Strains

*Mtb* H37Rv and HIV-1 strain JR-CSF were obtained from American Type Culture Collection (ATCC#27294, American Type Culture Collection, Chicago, IL, USA) and UCLA Center for AIDS Research, Los Angeles, CA, USA, respectively. Stocks of the strains were maintained at −80 °C.

### 2.4. In Vitro Macrophage Assays

RAW 264.7 murine macrophage cell line was cultivated in media described in Section 2.1 and plated in tissue culture 12-well plates at 0.5 × 10^6^ cells per well. Cells in respective wells were infected with Mtb (MOI 10.0) for 2 h to allow phagocytosis. The cells were washed with media to remove extracellular bacteria, replenished with fresh antibiotic-free media, and incubated in a CO_2_ (5%) incubator at 37 °C. Designated wells received 100 μM of nor-NOHA. After 48 h, cells were washed with PBS, lysed with 0.067% SDS, and 10-fold dilution of the lysate in sterile PBS was used for colony forming unit (CFU) enumeration by growth on 7H11 agar plates. The plates were incubated at 37 °C and CFU enumerated after a minimum of three weeks’ growth as we described [37]. Macrophages from matched wells were used to assess arginase activity, macrophage polarization, intracellular expression of arg1 and iNOS, and to identify metabolized products of arginine. Supernatants were collected to estimate nitric oxide produced.

### 2.5. Animal Infections and Treatments

*M. tuberculosis* H37Rv was propagated from growth stock in Middlebrook 7H9 media, (Cat#271310, Becton, Dickinson & Co., Sparks, MD, USA) [41] and used to deliver 200 colony forming unit (CFU) of Mtb for pulmonary infections. The required inoculum volume of bacterial suspension was diluted in PBS and delivered via intranasal (i.n.) route in 50 uL (25 μL per nare). For co-infection experiments, HIV from frozen stock was diluted in PBS. Mice were infected with 2500 TCID_50_ in 100 uL volume via the tail vein (i.v.) route. The timeline of infection, treatment with nor-NOHA (0.04 mg/g of mouse in 200 μL PBS) intraperitoneally (i.p.) daily, and organs collected for downstream analysis are detailed in figure legends. For CFU enumeration, the right lung (superior, inferior, and post-caval lung lobes) was collected in pre-weighed grinding tubes (Cat#35055A), Covidien, Cardinal Health 200, LLC, Waukegan, IL, USA) containing 1 mL sterile PBS. The lungs were homogenized and limiting dilutions in PBS were plated on 7H11 agar (Cat#BD 247940, Becton, Dickinson & Co., Sparks, MD, USA) plates as described previously [37]. Lung specimens were snap-frozen without buffer or with RNA*Later*^Tm^ stabilization solution (Cat#AM7024, life technologies, Eugene, OR, USA) for subsequent use to assess metabolites or gene expression, respectively, and stored at −80 °C. For immunohistochemistry (IHC), the left lung was preserved in 10% neutral buffered formalin (Cat#032-059 Fisherbrand, Pittsburg, PA, USA).

### 2.6. Nitrite (NO) Assay

Supernatants from lung homogenates were used to quantify NO from in vivo experiments as well as from RAW 264.7 in vitro. Supernatants were γ-irradiated (5MRAD) on dry ice to inactivate mycobacteria and facilitate work with samples at lower level of containment, as approved by the Institutional Biosafety Committee (IBC). Measure-iT^TM^ high-sensitivity nitrite assay kit (Cat#M36051, Molecular porbes, Life technologies, Eugene, OR, USA) was used to measure nitrite concentration as per manufacturer protocol and fluorescent intensity was measured using the BioTek Synergy H1 plate reader Agilent Technologies, Santa Clara, CA, USA. Nitrite concentrations were extrapolated using the linear regression of standards.

### 2.7. Arginase Activity Assay

Arginase activity was measured as amount of urea formed as a byproduct of conversion of arginine in whole-cell lysates of RAW 264.7 murine macrophage or lung tissue lysates according to a previously published protocol [42]. Briefly, cells were lysed in 0.1% triton containing a protease inhibitor cocktail (Cat #P8340), Sigma-aldrich, St. Louis, MO, USA) on ice for 30 min. An aliquot of the lysed cells (25 uL) was treated with MnCl_2_ (25 uL of 10 mM in 25 mM Tris-HCl) and incubated at 56 °C for 10 min for activation of arginase enzyme. L-arginine (100 μL of a 0.5 M in Tris-HCl, pH 9.7) was then added and the reaction mixture was incubated at 37 °C for 60 min for enzyme activity. The reaction was stopped using 400 μL of acid mixture (H_2_SO_4_:H_3_PO_4_:H_2_O; 1:3:7) followed by 40 μL of α-isonitrosopropiophenone (ISPF), (Cat # I3502, Sigma-aldrich, St. Louis, MO, USA) for development of color to measure formation of urea at an absorbance of 550 nm using a BioTek Synergy H1 plate reader. Enzyme activity was determined based on the amount of urea formed as extrapolated from urea standard concentrations and normalized to the amount of protein per sample.

### 2.8. Cytokine and Chemokine Quantification

Homogenized lung supernatants were inactivated via γ-irradiation (5MRAD) on dry ice, as described in [36], and confirmed to be free of viable Mtb following 3 weeks of growth on 7H11 agar plates. Chemokines and cytokines were identified and quantified using multiplex bead ELISAs from BioRad according to the manufacturer’s protocol. The Bio-Plex Pro Mouse 23-plex and the Bio-Plex Pro Human 40-plex (Bio-Rad, Hercules, CA, USA), assays were used to measure analytes in lung supernatants of Balb/cJ and HIS mice, respectively. Each molecule was quantitatively assessed using a standard curve generated from standards provided in the kit.

### 2.9. Assessment of Mϕ Polarization

To determine how the arginase inhibitor nor-NOHA altered in vitro macrophage polarization, RAW 264.7 cells were infected with Mtb in the presence or absence of nor-NOHA in a 12-well tissue culture plate using the experiment set-up described in Section 2.4. For flow cytometry experiments, GolgiStop (Cat#51-2092K2, BD Biosciences, San Jose, CA, USA) was added to all wells for the last 4 h of incubation to retain intracellular cytokines prior to staining. Cells were then collected in PBS followed by incubation with fixable viability marker (Cat#L10119, Molecular probes by Life Technologies, Eugene, OR, USA) for 10 min. Cells were then washed in FACS buffer containing 0.1% BSA and 0.1%NaN_3_ and incubated with an Fc receptor blocker for 5 min followed by addition of surface antibodies (diluted in BD Horizon^Tm^ Brilliant stain buffer, Cat#563794, BD Biosciences, San Jose, CA, USA) and incubated for 30 min at 4–8 °C. Surface antibodies used were CD206-BV650 (5 μL Cat#141723, Biolegend, San Diego, CA, USA) and CD80-BV711 (5 μL Cat#104743, Biolegend, San Diego, CA, USA). Fixation and permeabilization for intracellular staining were performed using the Cytofix/Cytoperm (Cat#2090KZ, BD Biosciences, San Jose, CA, USA) kit, followed by incubation with antibodies to intracellular molecules of interest (iNOS-APC (5 μL Cat# LS-C716205-100, LifeSpan, Shirley, MA, USA), IL-1β-FITC (2 μL Cat#11-7114-80, Molecular probes by Life Technologies, Eugene, OR, USA), arginase-1-eFluor 450 (2 μL Cat#48-3697-82, Thermo Fisher Scientific, Ward Hill, MA, USA), and IL-10-PE (5 uL Cat#505007, Biolegend, San Diego, CA, USA)) for 30 min. Samples were inactivated by incubation with 4% formaldehyde prepared from 16% ultrapure formaldehyde (Cat#18814-10, Polysciences, Warrington, PA, USA) for 48 h prior to acquisition.

Flow cytometry analysis was performed on a BD LSRII Fortessa cytometer BD Biosciences, San Jose, CA, USA) using stained cells and bead controls to develop a compensation matrix. Subsequent analysis was performed using FCS Express V6 software (de Novo Software, Inc., Pasadena, CA, USA). Side scatter and forward scatter characteristics along with viability dye uptake were used to select live cells for subsequent evaluation of a panel of M1 (iNOS, CD80, IL-1β) and M2 (Arg-1, CD206, IL-10) markers.

### 2.10. Histopathology and Fluorescent Immunocytochemistry

Paraffin embedding, sectioning (5 μM), and performance of hematoxylin and eosin (H&E) staining was performed at the UTMB Histopathology Core Facility. Immunofluorescent detection of arginase-1 and iNOS was achieved using multispectral staining, performed according to the manufacturer’s protocol, using the OPAL Multiplex Manual IHC kit. In brief, paraffin-embedded lung sections were placed in a 60 °C oven overnight followed by deparaffinization in in xylene (3 × 10 min) and rehydration through a series of decreasing ethanol gradients. Enzymatic and heat mediated antigen retrieval was performed using AR6 antigen retrieval buffer (Cat# AR600250ML, Akoya Biosciences, Hopkinton, MA, USA) in (3 × 5 min) EZ-Retriever microwave cycles at 95 °C. Blocking was performed using antibody diluent/block (Cat# ARD1001EA, Akoya Biosciences, Hopkinton, MA, USA) for 10 min prior to primary antibody incubation for arginase (Cat#ab96183, Abcam, Waltham, MA, USA) 1:100 overnight at 4 °C. After a series of rinses in TBST (2 × 3 min) OPAL 620 working solution (1:100) was incubated for 10 min at RT. Following rinses in TBST (2 × 3 min), the section underwent enzymatic and heat-mediated antigen retrieval and blocking as described above and was incubated with antibody to iNOS (Cat#abcam ab115819, Abcam, Waltham, MA, USA) 1:100 overnight at 4 °C. Slides were rinsed in TBST (2 × 3 min) and FITC conjugated secondary antibody (ABCAM ab6717) was incubated for 2 h at room temperature. Following rinses in TBST (2 × 3 min), nuclei were stained with SlowFade Diamond AntiFade mounting reagent with DAPI (4′, 6-diamidino-2-phenylindole) (Molecular probes by Life Technologies, Eugene, OR, Eugene, OR, USA). Whole-slide imaging was performed using the Hamatsu Nano Zoomer 2.0RS imaging system (Bridgewater, NJ, USA).

### 2.11. Metabolite Assessment Using LC-MS-MS

Metabolites were extracted in freshly prepared pre-cooled extraction solvent (methanol:10 mM PBS [80:20]). RAW 264.7 cells from tissue culture plates were collected, vigorously vortexed in the extraction solvent, and centrifuged at 14,000× *g* for 15 min. The supernatant was transferred to a fresh clean tube and 50 uL of isotopically labelled internal standards (ISs) corresponding to the endogenous metabolites was added to each sample. This mixture was mixed by vortexing and passed through a cellulose acetate membrane (Cat#8160, Costar, Corning Inc., Salt Lake City, UT, USA) twice to remove bacteria, and the filtrate was frozen at −20 °C until ready for analysis by mass spectrometry (MS). For extraction of metabolites from frozen tissue, pre-weighed tissue was homogenized using a bead-beater and remaining steps followed as for RAW 264.7 cells. A blank sample containing no cells or tissue was prepared in parallel as a control.

Amino acids and polyamines were analyzed via LC–MS-MS on an AB Sciex 6500 Q-trap mass spectrometer (Framingham, MA, USA) coupled with an Agilent 1260 ultra-high pressure liquid chromatography (UHPLC) system. The endogenous metabolites and their corresponding stable isotopically labelled internal standards (ISs) were separated by UHPLC on a Phenomenex Kinetex 5 μM ECO C18 (150 × 2.1 mm) column.

### 2.12. Statistical Analysis

Data were analyzed and graphed using GraphPad Prism 10 software and shown as the mean ± SEM. For multiple comparisons a one-way ANOVA was used. Comparisons between two groups was assessed using Student’s *t*-test. For all analyses, differences were considered significant at *p* < 0.05.

## 3. Results

### 3.1. Treatment with nor-NOHA Augments M1 Mϕ Phenotype and Antibacterial Activity of RAW Cells Infected with Mtb

**Figure 1:**
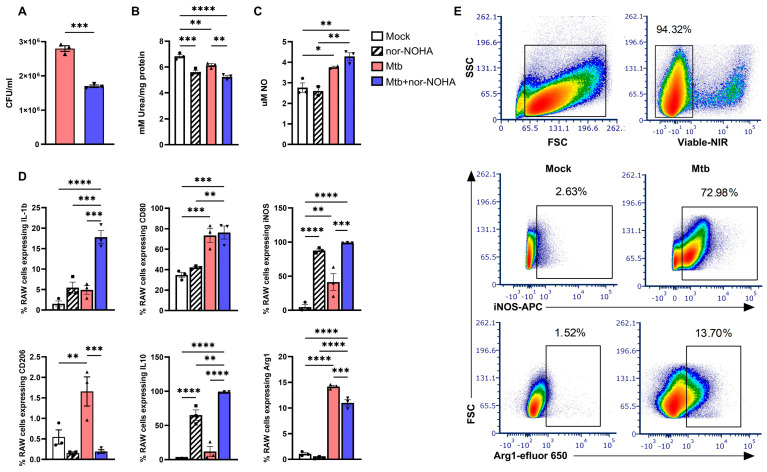
Treatment with nor-NOHA promotes M1 phenotype and antibacterial activity of murine Mϕ infected with Mtb. Macrophages were infected with 10 MOI of Mtb H37Rv for 48 h in the presence or absence of nor-NOHA followed by assessment of effects on (**A**) bacterial burden, (**B**) arginase activity, and (**C**) nitrite concentration. (**D**) Flow cytometric data of % of RAW 264.7 cells expressing CD80, IL-1β, NOS2, CD206, IL10, and arg1. (**E**) Gating strategy for flow cytometric analysis to determine viable cells that activate intracellular markers such as NOS2 and Arg1. Statistical analysis was performed using one-way ANOVA multiple comparisons. * *p* < 0.05, ** *p* < 0.01, *** *p* < 0.001, **** *p* < 0.0001.

As shown in Figure 1A, a significant decrease in mycobacterial growth was observed in the presence of nor-NOHA (10 μM) in murine macrophages. The inhibitor also decreased arginase activity (Figure 1B) as determined by the amount of urea formed in mock vs. nor-NOHA and Mtb vs. Mtb+nor-NOHA comparisons. The NO formed as a result of iNOS activity increased following Mtb infection. A further increase in NO was observed following nor-NOHA treatment of Mtb-infected cells, although this increase did not reach significance compared to Mtb infection (Figure 1C). Flow cytometry analysis was used to determine the number of cells producing arginase-1 and iNOS in association with other important M1 (CD80, IL-1β) and M2 (CD206 and IL-10) phenotypes (Figure 1D) among the live cell population as per the gating strategy (Figure 1E). Increases in cells that produce iNOS or Arg-1 were observed following Mtb infection (Figure 1D). Interestingly, treatment with nor-NOHA further increased the percentage of iNOS+ cells irrespective of Mtb infection. Treatment with nor-NOHA reduced the percentage of Arg-1+ cells following Mtb infection, although levels remained elevated compared to mock or nor-NOHA treatment controls. The response of other M1 and M2 phenotype markers varied with Mtb and nor-NOHA treatment. Mtb infection led to increased percentages of CD80, CD206, and a moderate, non-significant increase in IL-1β. Cellular IL-10 was activated by nor-NOHA treatment, an effect that was further increased by Mtb infection. The opposite pattern was observed for the CD206 marker, where treatment with nor-NOHA reduced the percentage. An increase in IL-1β+ cells was observed following treatment with nor-NOHA, an effect that was highly significant in cells infected with Mtb. The level of CD80 was not affected by nor-NOHA treatment in either mock or Mtb-infected cells.

**Figure 2:**
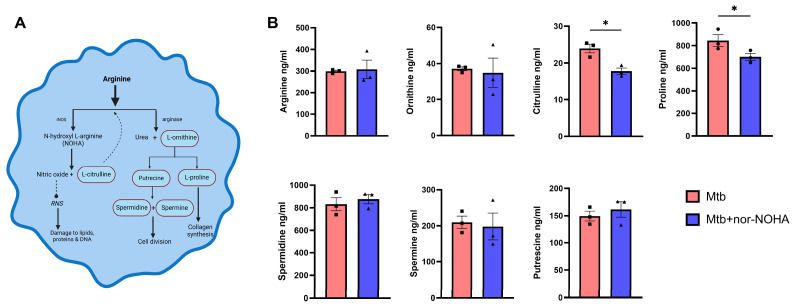
Metabolites downstream of arginine altered by nor-NOHA in murine macrophages infected with Mtb. Mϕ (RAW 264.7) were infected with 10 MOI of Mtb H37Rv for 24 h followed by assessment of effects on metabolites which were extracted in methanol/10 mM PBS [80:20] extraction buffer. (**A**) Arginine pathway within a macrophage. Image generated with permission using Biorender. (**B**) Concentration (ng/mL) of metabolites, amino acids (L-arginine, L-ornithine, proline, and citrulline), and polyamines (putrescine, spermidine, and spermine). Metabolite data shown are representative of three independent experiments of n = 3 per experiment. Statistical analysis was performed using the paired *t* test, two-tailed analysis. * *p* < 0.05.

The effects of in vitro arginase inhibition on the metabolites downstream of the L-arginine pathway (Figure 2A) were identified and quantified by LC-MS-MS. The concentration of L-arginine did not change in the presence of the inhibitor and downstream metabolic products of the arginase pathway also did not differ with the exception of proline. In contrast, the metabolic product of the competing iNOS pathway citrulline decreased in cells infected with Mtb and treated with nor-NOHA (Figure 2B).

### 3.2. Moderate Effects of nor-NOHA in Balb/cJ Mice Infected with Mtb

**Figure 3:**
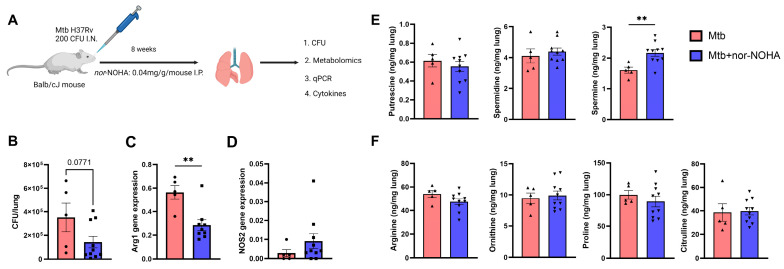
Sub-optimal outcomes of nor-NOHA on disease endpoints of Mtb-infected Balb/cJ mice correspond with limited effect on L-arginine pathway metabolites. (**A**) Timeline of infection and sample collection. Mice were infected with 200CFU of Mtb H37Rv via intranasal route, followed by treatment with nor-NOHA (0.04 mg/g mouse) i.p. daily for 8 wk. n = 5 for Mtb and n = 10 for Mtb+nor-NOHA. Mice were humanely euthanized at 8 weeks. Created with BioRender.com. (**B**) Altered bacterial growth, (**C**,**D**) arginase and NOS2 gene expression, (**E**) polyamines, and (**F**) amino acids in the lungs 8 weeks post-treatment with nor-NOHA compared to untreated. Polyamines and amino acids were extracted in extraction buffer methanol/10 mM PBS [80:20] from homogenized lungs. Statistical analysis was performed using the unpaired *t* test ** p < 0.01.

Balb/cJ mice infected with Mtb (200 CFU, i.n.) and treated with nor-NOHA (0.04 mg/g i.p.) daily for 8 weeks (Figure 3A) showed a trend (*p* = 0.077) towards a decrease in bacterial burden in the lungs (Figure 3B). This disease endpoint corresponded with a significant decrease in pulmonary arg1 and a non-significant increase in NOS2 gene expression in the presence of nor-NOHA (Figure 3C,D). The inhibitor had no effect on the concentration of metabolites involved in the arginase pathway, except for spermine (Figure 3E,F).

**Figure 4:**
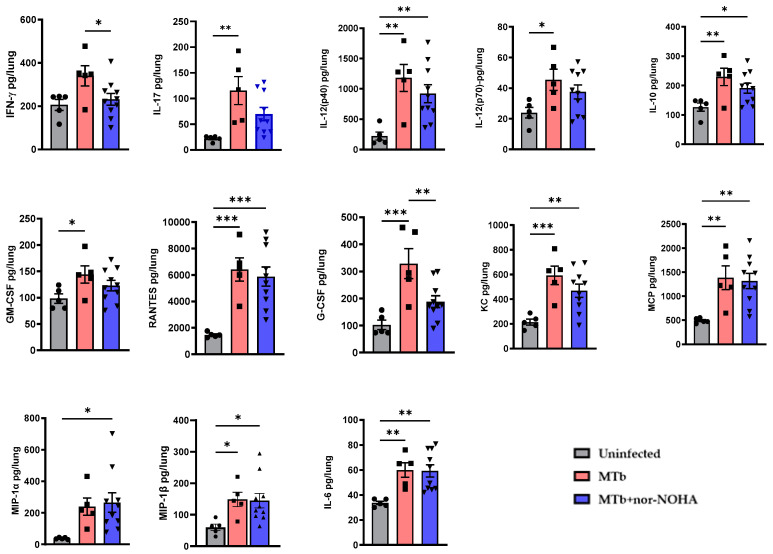
Therapeutic inhibition of arginase alters inflammatory cytokines in lung of Balb/cJ mice infected with Mtb H37Rv. Timeline of infection and sample collection as described in Figure 3A. Cytokine signatures measured using BioPlex Pro^TM^ mouse cytokine Grp 1 panel 23-Plex. Each cytokine was quantitatively assessed using a standard curve generated from standards provided in the kit. Statistical analysis was performed using ordinary one-way ANOVA for multiple comparisons. * *p* < 0.05, ** *p* < 0.01, *** *p* < 0.001. Additional cytokine and chemokine results without significance are shown in Supplemental Figure S1.

Assessment of soluble cytokines and chemokines in the lung of mice revealed that treatment with nor-NOHA alters some pulmonary immune responses to Mtb. In non-treated mice, several proinflammatory cytokines (e.g., KC, IL-6, MCP, GM-CSF, G-CSF, RANTES) and cytokines associated with T cell activity (e.g., IFN-γ, IL-17, IL-10, IL-12) increased as compared to non-infected animals (Figure 4). Other cytokines demonstrated a moderate increase (e.g., TNF-α, IL-1β, IL-1α) that did not reach significance due to variation among animals while no changes were observed for additional cytokines (e.g., IL-4, IL-5, IL-13, IL-9, IL-2, IL-3, Eotaxin) (Supplemental Figure S2). A similar activation of many cytokines was observed following treatment with nor-NOHA when compared to Mtb infection in the absence of nor-NOHA. Activation of MIP-1α and MIP-1β by Mtb, however, reached significance only when combined with nor-NOHA treatment (Figure 4). Interestingly, nor-NOHA treatment reduced the activation of IFN-γ and G-CSF by Mtb (Figure 4).

### 3.3. HIV Co-Infection Promotes Increased Arginase in the Mtb-Infected Lung

**Figure 5:**
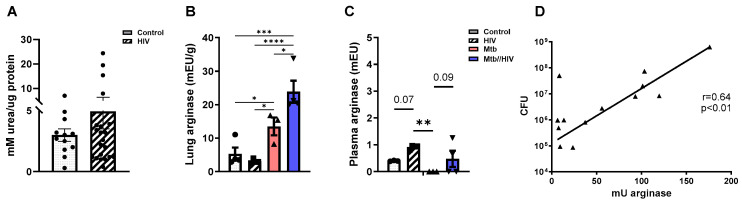
Pulmonary arginase is increased due to Mtb and HIV co-infection and correlates with bacterial burden (**A**) Pulmonary arginase levels in uninfected (control) vs. HIV-infected human lung specimens. (**B**,**C**) Altered arginase activity in plasma and lung of HIS mice. (**D**) Positive correlation between Mtb CFU with arginase in the lung. HIS mice were infected i.v. with HIV-1 JR-CSF (2500 TCID_50_) via tail vein and 3 weeks later a subset of mice were infected with Mtb H37Rv (tdTomato) 250 CFU intranasally. Arginase activity was measured in tissues 8 weeks post-infection with HIV and 5 weeks post-infection with Mtb. Statistical analysis was performed using the unpaired *t* test. * *p* < 0.05, ** *p* < 0.01, *** *p* < 0.001, **** *p* < 0.0001.

Elevated serum arginase has previously been described in the plasma of PWH [27,43]. Human lung tissue from PWH and age-matched controls obtained from the NNTC and both lung and plasma of HIV-infected HIS mice were used to expand these findings to the pulmonary compartment of relevant specimens and an experimental model. As shown in Figure 5A, assessment of arginase activity revealed no significant differences between HIV-positive and non-infected control human lung specimens. Similarly, HIV infection in HIS mice did not result in significant differences in pulmonary arginase (Figure 5B). Consistent with previous reports of human plasma, however, increased arginase was detected in plasma of HIV-infected humanized mice (Figure 5C). Co-infection of Mtb and HIV in HIS mice did not affect plasma arginase levels compared to HIV mono-infection (Figure 5C). Interestingly, pulmonary arginase demonstrated a further increase in animals co-infected with Mtb and HIV compared to Mtb mono-infection (Figure 5B). As shown in Figure 5D, pulmonary arginase levels were strongly and significantly correlated with Mtb CFU on a per lung basis (Figure 5D).

### 3.4. nor-NOHA Treatment Differentially Effects the Lung Cytokine Response of HIS Mice with Mtb and Mtb+ HIV in the Absence of Effects on Pathogen Burden

**Figure 6:**
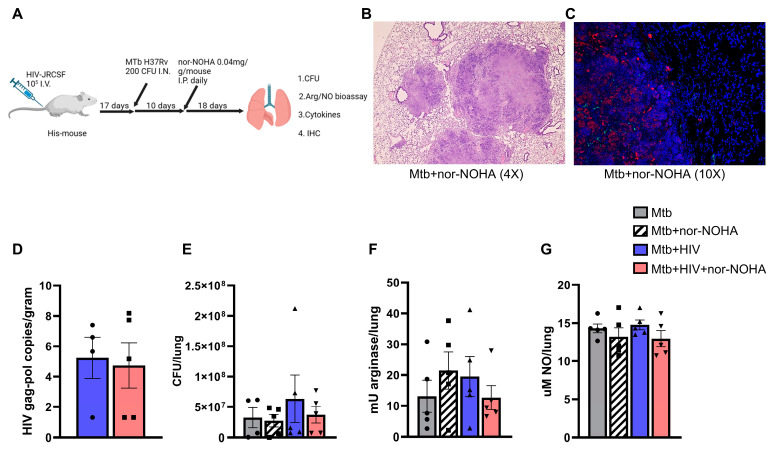
Disease outcomes in HIS mice with Mtb and Mtb/HIV coinfection are not altered by nor-NOHA treatment. (**A**) Timeline of infection followed by treatment with nor-NOHA; samples for various assessments collected 45 days post-infection and treatment with or without nor-NOHA n = 5. (**B**) H&E of lung granuloma. (**C**) IHC staining for arginase (red) and iNOS (green) treatment with nor-NOHA. (**D**) HIV gag-pol RNA copies (log) per gram of wet lung tissue. (**E**–**G**) Altered bacterial burden, arginase activity, and nitric oxide. Image in A created using Biorender and shared with permission.

To determine the potential to modulate TB disease outcomes in the setting of HIV co-infection using arginase inhibition, HIS mice were infected and treated with nor-NOHA according to the timeline shown in (Figure 6A). To demonstrate that HIS mice have the relevant L-arginine enzymes in lung granulomas (Figure 6B), specimens were used for histopathological and immune fluorescent imaging. Arginase (red) and iNOS (green) were visualized within and adjacent to TB granulomas in mouse lungs (Figure 6C). Treatment with nor-NOHA did not significantly alter disease outcomes, including in terms of viral copies (Figure 6D) and bacterial burden (Figure 6E) in animals experimentally infected with Mtb or Mtb and HIV. Consistent with these results, pulmonary arginase and iNOS activity were also unaffected (Figure 6F,G).

**Figure 7:**
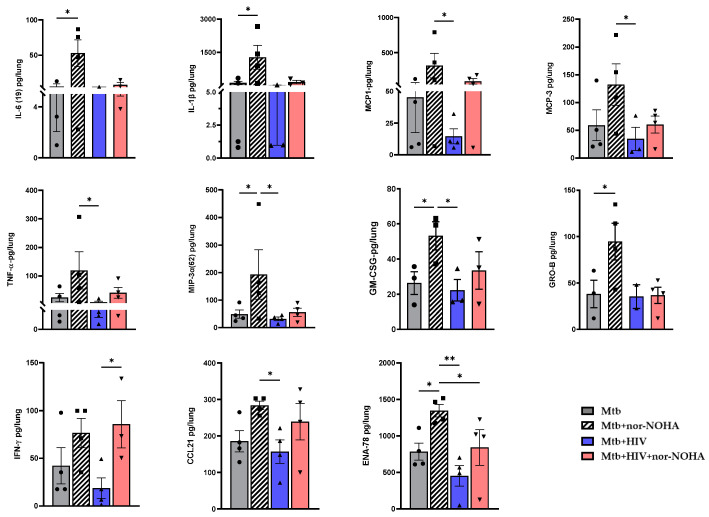
Therapeutic inhibition of arginase differentially alters pulmonary cytokine responses of HIS mice to Mtb and Mtb/HIV co-infection. Timeline of experiment as detailed in Figure 5A. Cytokines from lung tissue supernatants were measured using BioPlex Pro^TM^ human cytokine Grp 1 panel 40-Plex. Each cytokine was quantitatively assessed using a standard curve generated from standards provided in the kit. Statistical analysis was performed using one-way ANOVA, multiple comparisons, * *p* < 0.05, ** *p* < 0.005. Additional cytokine and chemokine results from the 40-Plex are shown in Supplemental Figure S2.

As we observed in BALB/cJ mice, treatment with nor-NOHA did promote some moderate changes in the cytokine response. Several cytokines in Mtb-infected mice were significantly increased due to nor-NOHA treatment (Figure 7), including IL-6, IL-1β, GM-CSF, GRO-B, and ENA-78, while the increase in other important cytokines (e.g., IFN-γ, TNF-α, MCP-1,3) did not reach significance (Supplemental Figure S1). In general, HIV co-infection led to a fairly similar production of cytokines when compared to the Mtb infection group. There were some notable differences between the Mtb and the Mtb+ HIV groups that were treated with nor-NOHA. Among mice treated with nor-NOHA (Figure 7), HIV co-infection significantly suppressed several human cytokines and chemokines, including MCP-1, MCP-3, TNF-α, MIP-3A, and GM-CSF. Interesting exceptions were IFN-γ and CCL21; treatment with nor-NOHA restored the cytokine response of co-infected mice to a level similar to the nor-NOHA-treated Mtb group.

## 4. Discussion

Host-directed therapies that activate antibacterial responses and limit inflammatory damage hold promise to improve pulmonary TB outcomes. Targeting host pathways that regulate antibacterial activity, promote tissue destruction (e.g., matrix metalloprotease or MMP), or modulate inflammation have shown some potential, as reviewed in [44,45]. The host myeloid cell L-arginine pathway is of interest for HDTs given the importance of NO in host defense against Mtb [46], the balance of NOS2 and Arg-1 associated with M1 macrophage antibacterial function [26], and the role of arginase in M2 Mϕ immune-regulatory and wound healing responses. Arginine, an essential modulator of cellular immune responses during many infections, including Mtb, is metabolized by the enzyme arginase, resulting in products such as ornithine and downstream products such as polyamines and L-proline [35,47]. The end products of the arginase pathway are also important in cellular processes like collagen synthesis, cell division and proliferation, gene expression for cell survival, regulation of apoptosis, and DNA and protein synthesis, etc. [48]. Our findings advance the field of TB HTDs by demonstrating how arginase inhibition by nor-NOHA alters Mϕ function and pulmonary disease outcomes in experimental models of TB and TB/HIV.

The in vitro effects of nor-NOHA treatment that we observed are promising with regard to modulating host macrophage arginine metabolism and promoting greater antibacterial activity. Treatment with nor-NOHA suppressed cellular arginase activity and increased NO production, as would be expected when limiting an enzyme such as arginase, which has competitive inhibition characteristics [49,50]. Consistent with the role of nitrosative stress for Mtb DNA damage [51], the increase in NO following arginase inhibition corresponded with a reduction in intracellular Mtb CFU. A reduction in Mtb CFU due to nor-NOHA treatment in vitro in a murine Mϕ cell line (Figure 1A) in our studies are similar to a report by Chakravortty D [34] for *Salmonella*. The importance of reactive nitrogen spp. in resistance to Mtb infection by two *inos* inhibitors (aminoguanidine and N^G^-monomethyl-L-arginine (NMMA) has also been shown, causing increased mortality, bacterial burden, and pathological tissue damage in Mtb-infected mice [25,52].

The in vivo efficacy of nor-NOHA in promoting antibacterial immunity in mouse models of pulmonary TB was limited, however, compared to the effects we observed in the cell culture. A trend (*p* = 0.08) towards reduced pulmonary Mtb burden in Balb/cJ mice was observed following 8 weeks of infection. The assessment of enzymatic activity in lung supernatants revealed a paradoxical increase in arginase activity and suppression of iNOS. Since nor-NOHA blocks the activity of de novo arginase, this outcome may reflect a feedback response whereby the host cell increases arginase. Additionally, there are multiple sources of arginase and iNOS in the lung that complicate interpretation. Therapeutic inhibition of arginase may impact several arginine metabolism pathways that function differently in diverse cell types [53,54]. In HIS mice infected with Mtb, with or without HIV co-infection, no difference in lung bacterial burden was observed following nor-NOHA treatment. There were no alterations in arginase or iNOS enzymatic activity in lung supernatants despite the conservation of arginase in murine and human systems and a previous demonstration that nor-NOHA is efficacious in a human cell [55]. The accelerated progression of Mtb infection in HIS mice [36] may be an important factor that limits the in vivo potential of arginase inhibition.

The differences observed between in vitro and in vivo outcomes may reflect the rapid clearance of nor-NOHA after treatment. A PK/PD study of nor-NOHA in Wistar rats injected via an i.p. route 30 mg/kg on day 1 or after five days of injection showed rapid clearance from plasma in approximately 90 min in both cases [56]. Deprivation of L-arginine led to increased iNOS without changes in NO production in murine Balb/c Mϕ exposed to *L. amazonesis*, as reported in [57] using an L-arginine-free medium. The short-lived forms of nitric oxide (NO, NO_2_, NO_3_^−^), typically in the range of 0.1–10 s, also contribute to difficulties in the accurate measurement of NO [58] in enzymatic assays (Figure 6G) that assess nitrites in cellular lysates as an estimate of NO. A recent report demonstrates the potential for nor-NOHA treatment to generate NO-like molecules in cell cultures and modulate some cellular outcomes [59]. These factors, along with other in vivo metabolism outcomes (e.g., liver processing), likely contribute to the differences in iNOS and arginase outcomes observed between cell lysates and lung supernatants when using enzymatic assays.

To develop a more comprehensive picture of how arginase inhibition with nor-NOHA alters the metabolism of arginine pathways, we identified and quantified metabolites in cell lysates and disrupted lung tissue by LC-MS-MS (Figure 2B). Based on the mechanism of action (inhibition of arginase activity), we expected to observe an increase in citrulline downstream of iNOS and reductions in ornithine, putrescine, proline, spermidine, and spermine downstream of arginase. The findings in both in vitro and in vivo systems demonstrate a disruption of the pathways, although the outcomes were unexpected. In murine cells (RAW Mϕ) infected with Mtb, both citrulline and proline were decreased following nor-NOHA treatment. Recycling of the citrulline formed in the NOS pathway in vitro via alternate pathways to arginine or other metabolites could be the reason for its decreased concentration [35]. Arginine pathway metabolites were mostly unchanged in Mtb-infected lung tissue due to daily nor-NOHA treatment. The exception was an increase in spermine (Figure 2B), a finding that supports the paradoxical increase in arginase activity that was observed (Figure 1B) in our study. Spermine is a polyamine that has recently been shown to mediate antimicrobial activity and enhance RIF and INH outcomes through generation of reactive oxygen species [60].

The effects of arginase inhibition by a different arginase inhibitor (N omega-hydroxy-D,L-indospicine) were shown to shift arginine metabolism to the iNOS pathway, but the shift was associated with changes in levels of iNOS through an unknown mechanism [61]. As a caveat, a comprehensive assessment of in vivo metabolic outcomes is challenging due to rapid changes in the metabolites in tissues. Additionally, alternative inputs and recycling of pathway metabolites further complicates interpretation of results. The timepoints chosen for sample collection are relevant to the measurement of immune outcomes as well as of the bacterial load. Effects at different timepoints during infection may also reveal earlier or later effects that are not apparent at the timepoints measured.

The results of both of our in vitro and in vivo experiments also demonstrate that nor-NOHA-mediated inhibition of arginase alters the immune response to Mtb infection. The role of Mϕ in disease outcomes is determined by the functional characteristics associated with polarization to specific subtypes such as M1, M2a, M2c, and others, as reviewed in [26]. Mtb infection is known to promote M1 polarization, which is characterized by abundant iNOS as well as increases in other M1 (e.g., CD80, IL-1β) molecules. The strong activation of innate immunity by Mtb also increases arginase-1 and other M2 (e.g., CD206, IL-10) markers in comparison to non-infected macrophages [26]. The inhibition of arginase promoted an even stronger M1 polarization of Mtb-infected RAW Mϕ, as indicated by the increases in intracellular NO and IL-1β and decreases in CD206 and Arg-1 that were detected using flow cytometry.

An especially interesting outcome was that inhibition of arginase produced a marked increase in IL-10 both non-infected and Mtb-infected macrophages. The effects on IL-10 may reflect a regulatory feedback response to excessive pro-inflammatory outcomes, such as the concomitant increase in IL-1β that is commonly observed including in the setting of Mtb infection [62,63]. IL-10 plays an important immune regulatory role including activation of anti-inflammatory genes that are important for limiting immune-mediated pathology in many infectious and non-infectious diseases (references). Autocrine IL-10 production plays a critical role in promoting the development of M2 macrophage polarization, which is linked with poor Mtb clearance in later stages of infection [64]. More recently, Arg2 was identified as one of the most prominent metabolic genes regulated by IL-10 in Mϕ, a pathway demonstrated to be key to regulation of bioenergetics in inflammatory Mϕs [65]. Our results suggest that inhibition of arginase impacts an important feedback loop whereby the disruption of the arginine pathway also activates greater IL-10. One potential mechanism could be through increased NO, a pro-inflammatory response that can activate IL-10 and other anti-inflammatory countermeasures [66,67,68]. However, the observation that IL-10 increased similarly in non-infected and Mtb-infected following nor-NOHA treatment suggests an alleviation of an IL-10 regulatory mechanism that is separate from NO metabolites. Alternatively, inhibition of arginase may affect IL-10 through other arginine metabolism pathways.

In the lungs of mice infected with Mtb, however, treatment with nor-NOHA did promote a stronger inflammatory cytokine response at the timepoint that was measured. Mtb infection has well established pro-inflammatory effects in the mouse lung and led to an expected activation of several molecules, including pro-inflammatory cytokines (e.g., KC, IL-6, MCP, GM-CSF, G-CSF, RANTES) and cytokines associated with T cell activity (e.g., IL-17, IL-10, IL-12) compared to non-infected animals. Other cytokines, IFN-γ, TNF-α, IL-1β, and IL-1α, increased moderately but did not reach significance due to variation, while no changes in IL-4, IL-5, IL-13, IL-9, IL-2, IL-3, and Eotaxin) (Figure 3 and Figure S2) were observed. In general, nor-NOHA treatment led to a moderate decrease in many cytokines in the lungs of Mtb-infected mice, although these effects only reached significance for IFN-γ and G-CSF. This is an interesting outcome given that IFN-γ pathway signaling is the predominant mechanism for the activation of iNOS to promote the catabolism of arginine to NO. These effects may reflect the moderate decrease in bacterial burden in the lung at this stage of infection and nor-NOHA treatment. The timing of measurements is also an important consideration for the comparison of in vitro results after 24 h with in vivo outcomes following 8 weeks of infection. During early infection, nor-NOHA may promote a stronger M1 polarization of lung Mϕ and a greater cytokine response, which promote greater antibacterial activity but also accelerate immune regulatory effects.

The effects of arginase inhibition using nor-NOHA also appear to differ from expectations based on arginase-deficient mouse strains. Our study represents the first described use, to our knowledge, of nor-NOHA in an animal model of TB. In other related studies, arginase 1 deficiency in mice led to alterations in amino acid metabolism, including increased citrulline and decreased proline and other branched-amino acids, while ornithine levels remained unaltered [69]. The importance of nitric oxide in the killing of mycobacteria was established in studies with IFN-ϒ and NOS2 knockout mice in which mice, when infected with Mtb, failed to produce reactive NO, resulting in a reduced ability to restrict the growth of intracellular bacteria [21,22,23]. These results demonstrate the importance of arginine metabolism via iNOS in order to promote NO-dependent intracellular Mtb growth restriction. Arginase and iNOS both compete for the substrate arginine. Several synthetic arginase inhibitors have been studied, with nor-NOHA showing promising results in animal and in vitro models of disease, including communicable and non-communicable diseases. nor-NOHA’s affinity to bind to active arginase sites has been reported to be in the nanomolar range [70]. Pharmacokinetically, it is highly bioavailable (close to 100%) after multiple doses and is rapidly eliminated t_½_ = 15–30 min after intravenous and intraperitoneal administration to rats [56,71]. However, it shows very poor pharmacokinetic profiles [30]. The arginase inhibitor nor-NOHA has been shown to delay leishmaniasis during Leishmania infection [72], modulate arginase activity during *Salmonella* infection [34], and affect T-cell dysfunction during injury generated by *L. monocyogenes* [73] and in human polymorphonuclear neutrophil granulocytes (PMNs) [74].

We further employed our HIS mouse model to explore outcomes of arginase inhibition in a humanized mouse model of TB, including in the setting of HIV co-infection. HIS mice develop granulomatous lung pathology following Mtb infection, as we have described [37] and further shown with H&E visualization in Figure 6B. In the current work, we further demonstrate the abundance of iNOS and arginase in areas of leukocyte organization in granulomas of Mtb-infected HIS mice (Figure 6C), the presence of which has been previously associated with organized zones of macrophages that are associated with inflammatory or regulatory phenotypes [75,76]. These observations, along with measurements of arginase and iNOS enzymatic end products in the lungs, indicate that that the lack of nor-NOHA efficacy in HIS mice was not due to a lack of these molecules. As previously mentioned, the accelerated course of disease may be an important factor as related to aggressive infection as well as shortened treatment course for nor-NOHA.

An important outcome we describe is that plasma arginase levels are increased in mice infected with HIV, consistent with increases in observed in peripheral blood mononuclear cells and plasma from HIV-seropositive patients [27]. Interestingly, we did not observe an increase in arginase in lung tissues of HIV-infected mice in the absence of Mtb. Utilizing preserved specimens of HIV-infected and control human lungs, we reproduced this result (Figure 5A) that demonstrates no significant change in arginase due to HIV in human lungs. Our observation suggest the potential for a tissue compartment effect of HIV to further exacerbate arginase, such as in lungs infected with Mtb. A caveat is that we did not have access to a human lung that was infected with both Mtb and HIV to validate the finding observed in the HIS mouse lung. Consistent with our observations, greater arginase activity was previously described in the blood of PWH who also suffered from visceral *Leishmaniasis* (VL) compared to HIV-infected controls [77]. Follow-up studies in VL patients demonstrated that IL-10- and TGF-β-dependent activation of arginase led to NO deficiency that was associated with disease presentation [78]. Collectively, these outcomes demonstrate the need to determine the cross-regulatory networks involving IL-10, NO, and arginase for the design of therapies that modulate arginine metabolism to augment immunity to Mtb or other infections.

## 5. Conclusions

These findings support further exploration of host-directed therapies that target arginase for use in preventive or treatment approaches for TB. Compounds with longer duration of action or other improvements in efficacy will likely be needed to conclusively determine their in vivo effectiveness. The use of nor-NOHA or other compounds that modulate arginine may have value as an adjunctive therapy to support antibiotic treatment or favorably modulate Mϕ polarization for therapeutic vaccination approaches. There are important safety concerns that would need to be addressed as well, the most important of which is the potential for immune-mediated pathology following the inhibition of a pathway that has a well-described role in modulating excessive inflammation. In the long term, a more comprehensive understanding of how the arginine metabolism pathway determines immune outcomes to Mtb at several timepoints is likely to be key in the development of novel interventions.

## Data Availability

The data presented in this study are openly available in Zenodo at 10.5281/zenodo.11495922.

## Supplementary Materials

The following supporting information can be downloaded at: https://www.mdpi.com/article/10.3390/tropicalmed9060129/s1. Figure S1. Altered inflammatory cytokines in lungs of Balb/cJ mice infected with Mtb H37Rv. Timeline of infection and sample collection as described in Figure 3A. Cytokine signatures measured using BioPlex Pro^TM^ mouse cytokine Grp 1 panel 23-Plex. Each cytokine was quantitatively assessed using a standard curve generated from standards provided in the kit. Figure S2. Inflammatory cytokine signatures in lung supernatants (without significance) of co-infected HIS mice (HIV/Mtb) treated with or without nor-NOHA. Timeline of experiment as detailed in Figure 5A. Cytokines from lung tissue supernatants were measured using BioPlex Pro^TM^ human cytokine Grp 1 panel 40-Plex. Each cytokine was quantitatively assessed using a standard curve generated from standards provided in the kit.
