# Supplementary material for: Therapeutic Modulation of Arginase with nor-NOHA Alters Immune Responses in Experimental Mouse Models of Pulmonary Tuberculosis including in the Setting of Human Immunodeficiency Virus (HIV) Co-Infection
Source: Trop Med Infect Dis. 2024 Jun 6;9(6):129. doi: 10.3390/tropicalmed9060129 (PMC11209148; doi:10.3390/tropicalmed9060129)
Supplement: Supplementary file 1 [file tropicalmed-09-00129-s001.zip › tropicalmed-2970996-supplementary.pdf]

Supplementary Materials:

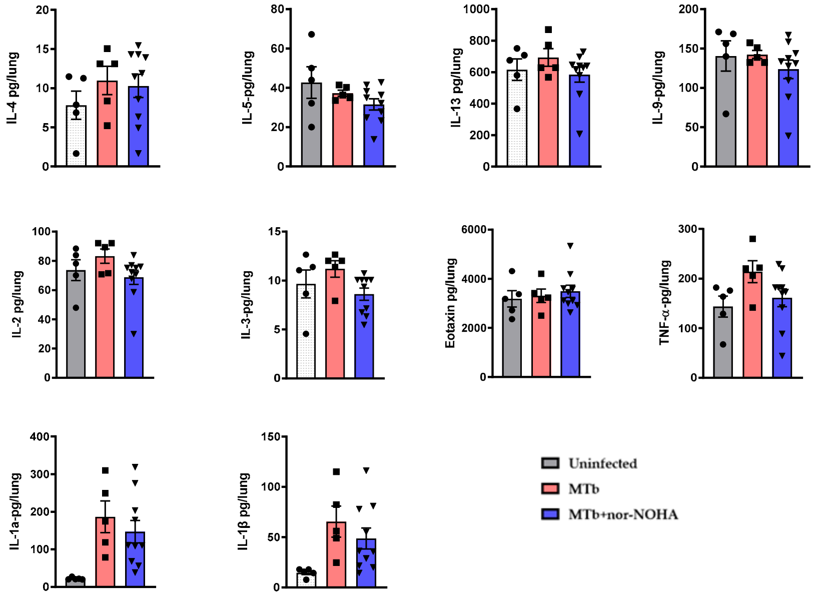

Figure S1. Altered inflammatory cytokines in lung of Balb/cJ mice infected with Mtb H37Rv. Timeline of infection and sample collection as described in Fig 3A. Cytokine signatures measured using BioPlex Pro™ mouse cytokine Grp 1 panel 23-Plex. Each cytokine was quantitatively assessed using a standard curve generated from standards provided in the kit.

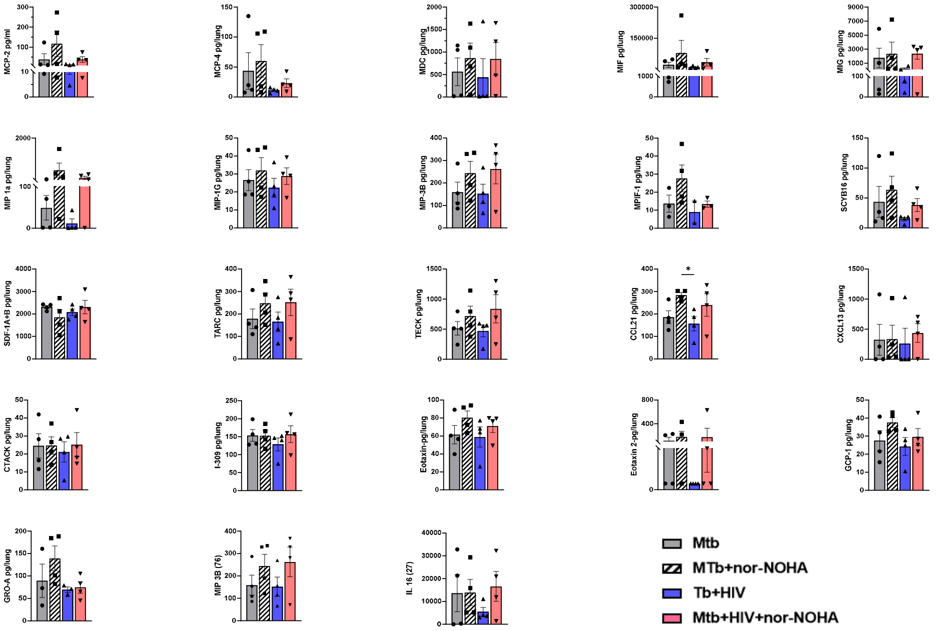

Figure S2. Inflammatory cytokine signatures in lung supernatants (without significance) of HIS-mice coinfected (HIV/Mtb) and treated with nor-NOHA or without. Timeline of experiment as detailed in Fig 5A. Cytokines from lung tissue supernatants were measured using BioPlex Pro™ human cytokine Grp 1 panel 40-Plex. Each cytokine was quantitatively assessed using a standard curve generated from standards provided in the kit.
